# Assessing the Impact of Family Planning Advice on Unmet Need and Contraceptive Use among Currently Married Women in Uttar Pradesh, India

**DOI:** 10.1371/journal.pone.0118584

**Published:** 2015-03-04

**Authors:** Diwakar Yadav, Preeti Dhillon

**Affiliations:** 1 Population Services International, Lucknow, Uttar Pradesh, India; 2 Save the Children, New Delhi, India; Peiking university third hospital, CHINA

## Abstract

**Background:**

Counseling/advice is one of the key interventions to promote family planning (FP) in developing countries, including India. It helps to improve the quality of care and reduce maternal deaths. This paper investigates the continuity of maternal health (MH) service utilization from antenatal care to post-natal care and the impact this service utilization has on contraceptive use and on meeting the demand for family planning among currently married women in rural Uttar Pradesh, India.

**Methods and Findings:**

The study assesses the impact of FP advice on unmet need and contraceptive use by adopting the propensity score matching method. It uses data from the District Level Household Survey (DLHS) (2007–08) that covered 76,147 currently married women (CMW) in the age group 15–44 years in Uttar Pradesh. Results show that the utilization of MH services [Antenatal care (ANC), institutional delivery, Postnatal care (PNC)] and FP advice during ANC and PNC has led to increase in current use of contraception by 3.7% (p<.01), 7.3% (p<.01) and 6.8% (p<.01), respectively. However, a greater utilization of these services has not translated into a reduction of unmet need for contraception at a similar manner.

**Conclusion:**

MH service utilization including FP advice is more effective in increasing current use of spacing methods as compared to limiting methods. Findings support the need for “effective FP advice” interventions to reduce unintended births and unmet need. However, women from Scheduled Caste/Scheduled Tribe communities are less likely to receive MH services. Thus, efforts are required to ensure that currently married women across socio-economic backgrounds have equal opportunity to receive MH services and information on contraceptive use to meet the demand for family planning methods.

## Introduction

Use of family planning (FP) methods is the main strategy for prevention of unwanted pregnancies [1,2]. It is estimated that almost 2,72,000 maternal deaths reported worldwide and 86,366 maternal deaths occurring in India every year could have been averted by the use of FP methods [3]. It was in the early 1950s that FP programs were first introduced in the developing countries to slow down population growth. Later in the 1980s global forums recommended that they be merged with maternal and child health (MCH) services for a more integrated approach [4]. In developing countries, where integration was a key element of the health system, birth rates have declined, as more women have been able to avoid unintended pregnancies[5]. To improve delivery, India has integrated MCH and FP services through the National Population Policy-2000, the National Health Policy-2002, the Reproductive and Child Health Program (Phase I-1997–2004, Phase II-2005–10) and the National Rural Health Mission (2005–12)[6–9]. Despite a plethora of policies and programs India is far from achieving its fertility goals. This is due to lower contraceptive use, high unmet need and unintended fertility [10].

The continuum of care including antenatal, natal and the post-natal care is critically important in high focus states of India. Here both mothers and children are vulnerable to a range of health risks resulting in high maternal and neonatal mortality. FP advice is an important component in the continuum of care. It is key to improving health behavior and health-care seeking during antenatal, natal and post-partum period [11]. The information provided during the antenatal care (ANC) enables women and their family members to later take care of their new born, adopt healthy behaviors and to identify and act on medical emergencies that may arise during antenatal, natal and post-natal care (PNC) periods [12,13]. Advice on appropriate FP methods ensures spacing between children and may contribute to preventing unwanted births. Although, previous studies show that FP advice has an inconsistent role in ANC service utilization and skilled birth attendance in the developing country settings [14–16].

Advice on FP is part of the standard practice of care for women who have just given birth. Demand for FP methods is potentially high after delivery and birth spacing plays a critical role in improving MCH [3,17]. Post integration most recent multi-country studies based on Demographic and Health Survey (DHS) data report a positive relationship between MCH service and contraceptive use [18–21]. This improvement is not explained by exogenous variables [22]. ANC services provide opportunities to reach women who would be the main target of FP services. This has been the rationale behind standard strategies using ANC as an entry point for the delivery of core reproductive health services, including FP [23].

Studies have shown that there is a preference among health workers for promoting ANC and institutional delivery while FP advice is very limited [24–27]. Earlier research suggests that an ANC package including FP advice significantly increased the quality of care[28]. In South Asia region [29–31] particularly in India, studies show that PNC is limited to inequality in service utilization [32–34].

Studies examining the value of integration have mixed findings. A randomized control trial on educational interventions for contraceptive use reveals that women who received postpartum counseling with repeated contacts were more influenced to use FP methods[35]. However a review of trials found that there was little impact of integrated service delivery on outcomes of integration, costs or health system performance in developing country settings[36]. Two other trials showed that integration of FP service delivery resulted in increased contraceptive use [37,38].

Being most populous state and having the low socio-economic status, Uttar Pradesh is high focused state with 25 (19 administrative districts by Government of India and additional 6 administrative districts by state Government of Uttar Pradesh) out of 70 administrative districts in 184 high priority districts in India under NRHM[9]. Although, NRHM was launched in 2005 with an integration approach, there is a dearth of literature particularly in Uttar Pradesh identify the role of MCH care in increase in contraceptive use and decrease in unmet need for FP. Therefore, the present study is an attempt to investigate the continuity of service utilization from ANC to contraceptive use in a rural setting of Uttar Pradesh. This paper mainly assesses the role of FP advice provided as part of MH services, in increasing the use of contraceptives and reducing the unmet need for FP among currently married women (CMW). This study of MH service utilization and particularly advice during ANC and PNC sessions would be crucial for strengthening such public health service program efforts further.

## Materials and Methods

### Ethical statement

All respondents in this study provided written informed consent. The District Level Household and Facility Survey (DLHS) protocol and ethical clearance were obtained from the Ethics Committee of International Institute for Population Sciences, Mumbai and the Ministry of Health and Family Welfare, Government of India, New Delhi. The DLHS dataset is available in the public domain for research and no formal approval from the institution is required. In addition, the survey instrument is available on the DLHS website (www.rchiips.org).

### Data and study settings

The present study utilises data from the third wave of DLHS (2007–08) that covered all 601 administrative districts from 34 states and union territories of India. This survey provides estimates on MCH services and FP use at the district level in order to monitor and provide corrective measures to the NRHM. The survey adopted a multi-stage stratified sampling design and used a set of structured questionnaires for collection of data [39].

The present work focuses on rural population of Uttar Pradesh (UP). UP constitutes 18.6% of the total rural population of India, one of the largest in the country [40]. It is significantly diverse in its socioeconomic, demographic, geographic and cultural profile[10]. It is currently passing through the third stage of demographic transition, with an estimated death rate of 8.2 per thousand population and infant mortality rate of 63 per thousand births[41]. A large proportion of the state’s population suffers from poverty and fares poorly on indicators of gender equality such as female literacy and women’s autonomy. In the Human Development Index, it ranked 13^th^among 15 major states of India[42].

The trends in service utilization of MH and contraceptive use in Uttar Pradesh do not reflect much improvement over the last two decades [10]. The recent Indian Demographic Health Survey (2005–06) for Uttar Pradesh reveals that a mere fourth (27%) of the pregnant women made 3+antenatal visits during their most recent pregnancy. Only about a fifth (21%) had their most recent deliveries in a health facility and only 15% of the women reported receiving PNC after their most recent birth. Moreover, only 29% of women were using any modern contraceptives and unmet need for limiting methods (21%) is higher than that for spacing methods (9%).

In DLHS-III, the information was gathered from a representative sample of 90, 415 households, 87,564 ever-married women (aged 15–49 years) and 76, 147 CMW (aged 15–44 years) of 70 administrative districts in Uttar Pradesh. The study sample considered 31, 865 CMW in the age group 15–44, who had given birth during the reference period. Data on antenatal, natal, post-natal care was collected from all women who had given birth in the five years preceding the survey and were restricted to the most recent birth. The household and ever married women response rates were 94% and 84%, respectively. Appropriate weights given in the data are used.

### Outcome variables

The study considers two outcome variables, contraceptive use and unmet need for FP defined as:

**Current use of modern FP methods**. Information was obtained from the CMW by asking them the question “whether you or your husband are currently using any FP methods (Yes/No)?” Those who responded in the affirmative, were further asked about name of the method. Contraceptive prevalence rate (CPR) for spacing method is defined as the percentage of CMW themselves or their husbands using an intrauterine device, oral pills, condoms, injectibles, foam or jelly and implants on the date of the survey (coded as Yes-current use and No-no use). CPR for the limiting method is defined as the percentage of CMW themselves or their husband using sterilization on the date of the survey (coded as Yes-current use and No-no use). Total current use of modern contraceptives includes the current use of both spacing and limiting methods (coded as ‘Yes’) versus not using modern contraceptive methods (coded as ‘No’).

**Unmet need for modern FP methods**. *Unmet need for spacing methods* includes the proportion of CMW who are neither in menopause nor a hysterectomy nor are currently pregnant, wanting more children after two years or later and are currently not using any FP method. The CMW who are unsure about having another child are also included here and so are those still intending to have one but are unsure of the timing. *Unmet need for limiting methods* includes the proportion of CMW who are neither in menopause nor had a hysterectomy nor are currently pregnant and do not want any more children but are currently not using any FP method. Total unmet need refers to unmet need for limiting and spacing. Unmet need for FP variable was already given in data file, so we used the same variable and dichotomized as 1-unmet need for FP and 0-met need for FP.

### Exposure variables

The study considers three critical services namely-ANC visits (Yes/No), institutional delivery(Yes/No) and PNC within two weeks of delivery (Yes/No) in MH service utilization. Further, women who reported using/attending all three critical services are considered as having received all MH services (coded as ‘Yes’), a proxy for exposure to the MH program and the remaining as unexposed (coded as ‘No’).

The study considers the advice during ANC and PNC session as exposure to the MH program. Detailed information on advice received is as follows-Women were asked if they had received advice on seven essential components of specific MH care services during the ANC visit. These components include: (1) breastfeeding (Yes/No), (2) keeping the baby warm(Yes/No), (3) importance of cleanliness at the time of delivery(Yes/No), (4) nutrition for mother and child (Yes/No), (5) importance of institutional delivery (Yes/No), (6) spacing methods of FP (Yes/No) and (7) limiting methods of FP (Yes/No). The exposure variable used in the analysis is the FP advice received during ANC visits, including spacing and limiting methods of FP (coded as ‘Yes’) and the remaining as unexposed (coded as ‘No’).

During the PNC visit, women were asked if they had received advice on four essential components of MH care specific services including (1) abdominal examination (Yes/No), (2) advice on breastfeeding (Yes/No), (3) advice on infant care (Yes/No) and (4) advice on FP methods (Yes/No). The exposure variable FP advice received during PNC visit (coded as ‘Yes’) and the remaining as unexposed (coded as ‘No’) was used in the analysis.

### Explanatory variables

Socioeconomic and demographic predictors such as the woman’s age, education, children ever born, religion, ethnicity-caste and household wealth quintiles were included in this study. The women’s ages were categorized into less than 25 years and 25 years or above. The educational level of women was defined using years of schooling and was categorized into less than 5 years of schooling, 5–9 years of schooling and 10 years of schooling or above. The population was assigned to two religious categories Hindu and non-Hindu (Muslim, Jain, Sikh, Christian and others), because disaggregation of minorities had little statistical significance given their small numbers. The sample was categorized into: Scheduled Castes (SC)/Scheduled Tribes (ST), Other Backward Castes (OBC) and ‘Others’. This nomenclature has used the terminology adopted by the Government of India, focusing more on the socially disadvantaged castes/tribes while all privileged social groups are classified as ‘Others’.

In the absence of direct information on income in household surveys like the DLHS, the wealth index is widely used as a proxy indicator for assessing the household economic status. In developing countries the index has been found to correlate highly with income data [43,44]. The DLHS collects a whole range of information on consumer durables, housing conditions, water and sanitation facilities- used as a proxy for household economic status. In the third wave of the DLHS, the wealth index was created using principal component analysis (PCA) on items related to possession of durable assets, access to utilities and infrastructure and housing characteristics. The PCA scores in the dataset were weighted by the household sampling weights to ensure that the distribution was representative of all households in Uttar Pradesh following which the households were divided into quintiles. A detailed description on the methodology adopted to construct the wealth index in DLHS dataset is provided in the DLHS-3 national report [39].

### Analytical approach

Bivariate analysis was conducted to assess the continuum of service utilization. The proportion of CMW using two or more consecutive services in a sequential manner as provided by the health system are analysed. The percentage of those utilising two consecutive services in the sequence in which they are dispensed by the health service system are considered as those who have utilised a continuum of service. The complement of this percentage provides the dropout. The Chi-square test is applied to examine the association between services in the bivariate analysis. All tests are two tailed and a p-value of <0.05 is considered statistically significant.

In order to examine the impact of FP advice received during ANC/PNC sessions and MH care service utilization on unmet need and current use of modern FP methods, the study adopted the Propensity Score Matching (PSM) [45,46]. This approach gives an opportunity to assess the impact of exposure on program outcomes through cross-sectional survey data [47–49]. The study used radius caliper method for matching that reduces the risk of using poor matches as it uses all the possible comparison group members within the maximum distance from caliper [45,50]. The common support restriction (to exclude data from exposed with a propensity score higher than that of any unexposed person) was imposed to improve the quality of matching.

The propensity score is estimated by logistic regression, with the dichotomous exposure/treatment variable. For instance, 1 = exposed to FP advice during ANC; 0 = unexposed to FP advice during ANC. Associated observed selected characteristics of the CMW and household namely women’s age, education, children ever born, religion, caste and wealth index are used as predictor variables.

There are two key assumptions in PSM procedure; first, conditional independence assumption i.e. assignment to exposed and unexposed can be considered as random after controlling covariates. We checked this assumption by using <pscore> and <ptest> commands, former command examines the balancing property which states that, conditional on the propensity scores, the distribution of confounding factors are similar among exposed and matched unexposed and later gives covariate imbalance testing i.e., t test (mean) and χ2 test (percentage) results to check the differences in background characteristics of exposed with (matched) unexposed individuals. Second assumption under PSM is on common support condition i.e. for each value of covariates there is a positive probability of being exposed or unexposed. Used command <pscore> also gives information regarding common support area. We used <psgraph> for common support area graphing. All analysis was done using STATA (version 11), and we used <psmatch2> package for PSM method[51].

PSM method explained to evaluate programs impacts have been recently facilitated by improvements in computing capacity and associated algorithms and matching approach[47]. In recent past, selected studies have carried out using this method to evaluate public health related programs in India[50,52–55]. These have shown a significant effect of the intervention programs on HIV related risk among the highly risk groups such as female sex workers[53] and truck drivers[54].

In this case, difference in unmet need and current use of modern FP methods between exposed and control groups can be directly compared to show the impact of exposure on the exposed group. This is known as average treatment effect on those treated (ATT). Additionally, comparing the difference in unmet need and current use of modern FP methods between control and matched exposed groups can show the impact of exposure on the unexposed group. This is known as average treatment effect on the untreated (ATU). These two average effects were weighted by the proportion of women in exposed and control groups, respectively, to arrive at the impact of the service received on unmet need and current use of modern FP, known as average treatment effect (ATE). This measured the change in unmet need and current use of FP due to FP advice and maternal health care service utilization.

Exposed CMW and the matched control CMW were compared on parameters of unmet need and current use of modern FP methods to examine the impact of FP advice. To assess whether the average effect is statistically significant, bootstrapped SE around the estimates[56,57] is calculated. The study has used STATA 11.0[51] package for the entire analysis.

## Results

### Continuum of maternal health service utilization and contraceptive use

**Fig 1:**
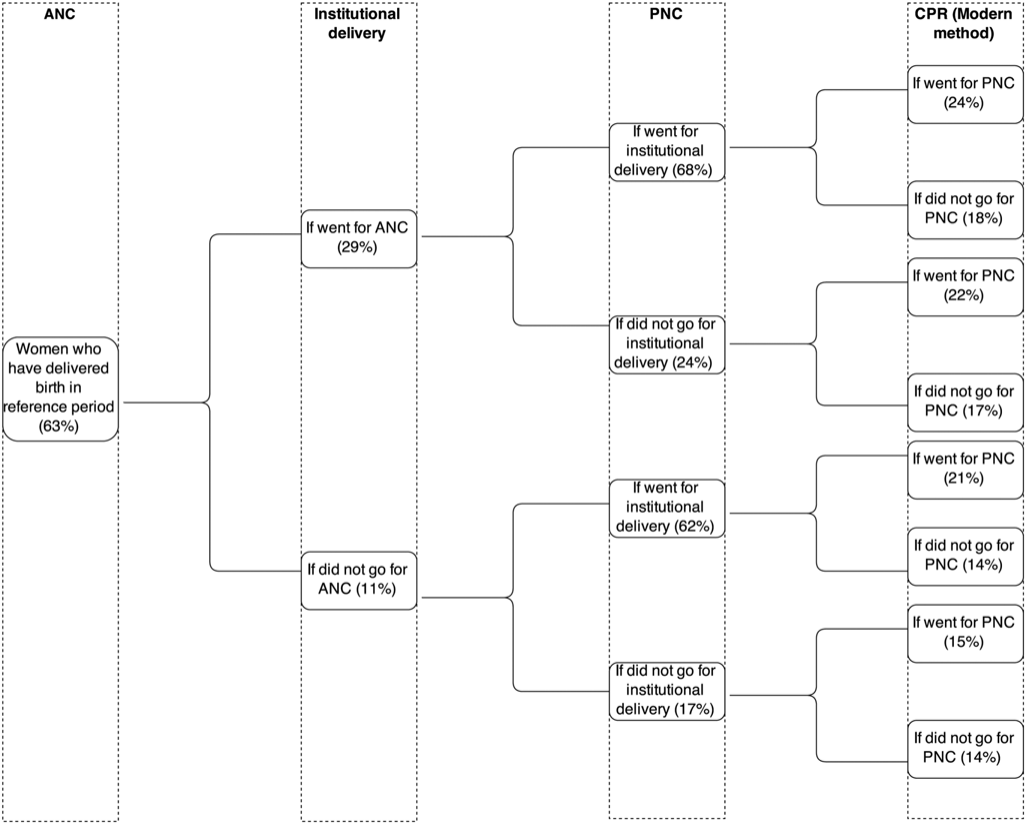
Flow chart of process of service utilisation at different levels in rural Uttar Pradesh, 2007–08.

Fig. 1 shows the continuum of MH service utilization and contraceptive use in rural Uttar Pradesh at different stages of service provision. Nearly 63% CMW have received any ANC. Out of those only 29% have delivered their babies in institutions compared to 11% of those who did not receive any ANC.

Among the CMW who received both prior services, 68% have availed PNC services as compared to 24% among those who received any ANC and skipped institutional delivery. The proportion of CMW who did not utilise any ANC but had adopted institutional deliveries and received PNC services is 62%. Only 17% of women who did not avail both ANC and institutional delivery utilised PNC services. Of the CMW who received all these three critical services 24% are currently using modern contraceptive methods in contrast to 14.0% among who have not availed any MH services. It is evident from this analysis that the utilization of a service is more likely if the woman avails the service provided prior to it as part of the continuum of MH services. The continuum of MH service utilization also encourages contraceptive use.

### Levels of maternal health service utilization by background characteristics of women

The mean age of CMW interviewed was 26.8 years. Of the 31865 CMW, nearly 70% had less than 5 years of school education, 84% were Hindu, 25% belonged to SC/ST categories and 26% were in the poorest wealth quintile. A total of 3905 (12.3%) CMWs utilized all maternal health service namely- any ANC, had institutional deliveries and received PNC. CMW aged below 25 years (15%), with 10 or more years of education (38%), were Hindu (13%), belonging to non-SC/ST/OBC (22%) categories and from the richest wealth quintile of households (37%) had utilised all MH services (Table 1).

**Table 1:** Proportion of currently married women who used maternal health services and received advice by background characteristics (Uttar Pradesh, India; DLHS, 2007–08).

Women’s background characteristics	Total n (%)	All maternal health service utilisation* (%)	Family planning advice received during ANC sessions (%)	Family planning advice received during PNC sessions (%)
**N**	31865	3905 (12.3)	4495 (14.1)	2396 (7.5)
**Age**				
**Mean age in years (SD)**	26.8 (5.8)	25.3 (5.0)	26.2 (5.3)	26.1 (5.3)
**Less than 25 years**	12721 (39.9)	15.3	15.2	8.4
**25 years or more**	19146 (60.1)	10.2	13.5	7.1
**Education**				
**Less than 5 years education**	22168 (69.5)	7.3	9.6	4.9
**5–9 years education**	6783 (21.3)	17.4	19.3	10.1
**10 years and above**	2916 (9.2)	37.6	37.1	22.1
**Religion**				
**Hindu**	26885 (84.4)	12.6	15.0	7.8
**Non-Hindu**	4982 (15.6)	10.5	9.8	6.4
**Caste**				
**SC/ST**	7905 (24.8)	8.1	12.2	5.5
**OBC**	18226 (57.2)	11.1	12.9	6.7
**Others**	5736 (18.0)	21.8	21.1	13.2
**Wealth Quintile**				
**Poorest**	8410 (26.4)	5.1	8.0	3.6
**Second**	7681 (24.1)	7.8	10.8	5.3
**Middle**	6896 (21.6)	10.8	13.7	7.0
**Fourth**	5778 (18.1)	17.1	18.6	10.0
**Richest**	3102 (9.7)	37.0	32.1	21.2

*all maternal health service utilisation includes: received any ANC service, had institutional delivery, and received postnatal care check-up.

Only 4495 (14%) and 2396 (7.5%) CMWs had received FP advice during ANC and PNC session, respectively. The advice on FP during ANC and PNC sessions were received more among CMW who had education of 10 years or more, were non-SC/ST/OBC and from households in the richest wealth quintile.

### Unmet need and contraceptive use by different maternal health service utilization

The current use of modern contraceptive methods among CMW who received all MH services (any ANC, Institutional delivery, PNC) in 2007–08 is 24% in contrast to 17% among those who did not receive these services. The proportion of CMW using modern contraceptive methods was higher, at 27% if they received advice during ANC visit and still more at 29% among those who were counselled during the PNC visit. These bivariate results reveal that advice on FP particularly during PNC has motivated CMW to use modern contraceptive methods. PNC has had a 12% effect on the increase of current use of FP. However, the CMW’s characteristics were not controlled in this analysis.

16% and 9% of those who received all MH services use spacing and limiting methods respectively as compared to only about 8% and 9% among those who have not received such services. Of those who received advice during ANC visits 16% and 11% used spacing and limiting methods respectively, while the figures are 18% and 11% among those who have received advice during PNC. These findings suggest MH services utilization has influenced FP use particularly spacing methods. Thus advice during ANC and PNC has been effective in increasing CPR for the spacing method by 8% (p<0.01) and 9% (p<0.01) respectively.

The paper highlights the effect of MH service utilization on reducing unmet need for FP through bivariate analysis as shown in Table 2. It reveals that about 35% of CMW who have received all MH services have unmet need for FP in comparison to 39% who have not received such services. Nearly 34% of the CMW who have received FP advice during ANC or PNC reported unmet need for FP. These results reveal that all MH service utilization and FP advice has marginal effect on unmet need for FP. However, of the two FP methods utilization of MH services and advice during ANC or PNC visits have greater impact on reducing unmet for limiting methods. MH service utilization has a higher impact on increasing use of modern methods; however, these are not sufficient for reducing unmet need for FP.

**Table 2:** Current use of contraceptives and unmet need for family planning methods by selected maternal health service utilisation (Uttar Pradesh, India; DLHS, 2007–08).

MH Service Utilisation variables	Outcome variables					
	Current use of contraceptives			Unmet need for family planning methods		
	Total	Spacing	Limiting	Total	Spacing	Limiting
**ANC, ID & PNC**						
**No**	16.5	8.0	8.5	38.6	10.7	27.8
**Yes**	24.2	15.7	8.5	34.8	13.7	21.1
**Difference**	7.7^†^	7.7^†^	0.0^€^	-3.8^†^	3.0^†^	-6.7^†^
**Advice received on family planning methods during ANC visits**						
**No**	15.9	7.8	8.1	38.8	11.2	27.6
**Yes**	26.7	15.9	10.9	33.9	10.4	23.5
**Difference**	10.8^†^	8.1^†^	2.8^†^	-4.9^†^	-0.8^††^	-4.1
**Advice received on spacing methods of family planning during ANC visits**						
**No**	-	7.9	-	-	11.2	-
**Yes**	-	16.8	-	-	10.5	-
**Difference**	-	8.9^†^	-	-	-0.7^€^	-
**Advice received on limiting methods of family planning during ANC visits**						
**No**	-	-	8.1	-	-	27.5
**Yes**	-	-	11.3	-	-	23.1
**Difference**	-	-	3.2^†^	-	-	-4.4^†^
**Advice received on family planning methods during PNC visits**						
**No**	16.5	8.2	8.3	38.5	11.1	27.4
**Yes**	28.5	17.6	10.9	34.2	11.2	23.0
**Difference**	12^†^	9.4^†^	2.6^†^	-4.3^†^	0.1^†^	-4.4^†^

Note: ANC, antenatal check-up; ID, Institutional delivery; PNC, postnatal check-up; χ2 test are performed.

significance level:

^†^p<0.01,

^††^p<0.05,

^€^p<0.10

### Impact of advice received on contraceptive use and unmet need for family planning

The study estimates the impact of service utilization on current use of contraceptive and unmet need for FP by the estimated difference in both the outcomes, between the treated (receivers) and the matched control (non-receivers) groups. The utility of this matching analysis is that one gets to see the actual impact of the treatment as it controls background variables as well as the characteristics of women who were not treated or did not participate. This latter could be the result of selected women receiving services or the health provider’s selection bias. An Indian study using the same data set reveals that women from lower socio-economic backgrounds are less likely to receive MCH advice[58]. The effect of the program on the group who did not participate, that is, if they could have participated what would be the effect on outcome, is given.

Results from Table 3, the average treatment effect (ATE) of all MH service utilization on current use of contraception is 3.7% (p<0.01). The ATEs of receiving FP advice during ANC and PNC visit are 7.3% (p<0.01) and 6.8% (p<0.01), respectively. This slightly higher effect of ANC is due to the higher effect on the untreated group, i.e. the effect when the women who did not go could have gone for ANC. On the other hand, ATE of utilization of all MH services on reducing unmet need for FP is only 0.5%. Receiving advice during ANC and PNC have led to reduction in unmet need by 3.1% and 1.4% points, respectively. The ATEs of exposure to MH services by type of method are estimated consistently higher for spacing methods. ATEs of availing all MH services are 2.9% (spacing) and 0.8% (limiting), of receiving FP advice during ANC were 4.0% (spacing) and 3.3% (limiting), and of receiving advice during PNC are 3.6% (spacing) and 3.2% (limiting).

**Table 3:** Estimated impact of advice on contraceptive use and unmet need for family planning methods among currently married women.

Treatment variables	Outcome variables	Treated^#^ (%)	Untreated^$^ (%)	ATT (%)	ATU (%)	ATE (%)
**MH utilisation (ANC, ID & PNC)**	Current use of modern methods	24.3	20.4	3.8	3.7	3.7^†^
	Current use of spacing methods	15.8	12.4	3.4	2.8	2.9^†^
	Current use of limiting methods	8.5	8.0	0.4	0.8	0.8^†^
	Unmet need for modern methods	34.7	35.1	-0.3	-0.6	-0.5^†^
	Unmet need for spacing methods	13.6	11.9	1.6	1.5	1.5^†^
	Unmet need for limiting methods	21.2	23.1	-1.9	-2.1	-2.0^†^
**FP advice received during ANC sessions**	Current use of modern methods	26.7	19.9	6.8	7.4	7.3^†^
	Current use of spacing methods	15.9	11.8	4.1	4.0	4.0^†^
	Current use of limiting methods	10.8	8.1	2.7	3.4	3.3^†^
	Unmet need for modern methods	33.9	36.5	-2.5	-3.2	-3.1^†^
	Unmet need for spacing methods	10.5	11.7	-1.3	-1.7	-1.6^††^
	Unmet need for limiting methods	23.5	24.8	-1.3	-1.5	-1.5
**FP (spacing) advice received during ANC sessions**	Current use of spacing methods	16.8	12.3	4.5	4.4	4.4^†^
	Unmet need for spacing methods	10.6	11.9	-1.3	-1.6	-1.5^€^
**FP (limiting) advice received during ANC sessions**	Current use of limiting methods	11.2	8.2	3.1	4.1	4.0^†^
	Unmet need for limiting methods	23.1	24.4	-1.3	-1.8	-1.7^†^
**FP advice received during PNC sessions**	Current use of modern methods	28.6	20.8	7.8	6.8	6.8^†^
	Current use of spacing methods	17.7	12.9	4.7	3.5	3.6^†^
	Current use of limiting methods	10.9	7.9	3.0	3.2	3.2^†^
	Unmet need for modern methods	34.2	36.1	-1.8	-1.3	-1.4^†^
	Unmet need for spacing methods	11.2	11.7	-0.5	-0.6	-0.6^†^
	Unmet need for limiting methods	23.0	24.3	-1.4	-0.7	-0.8^†^

^#^Treated (exposed to treatment variable).

^$^Untreated (Unexposed to treatment variable).

ATT- Average treatment effect among treated; ATU- Average treatment effect among untreated; ATE- Average treatment effect; ANC, antenatal check-up; ID, Institutional delivery; PNC, postnatal check-up.

Significance level:

^†^p<0.01,

^††^p<0.05,

^€^p<0.10

The overall message from this analysis is that the MH service utilization including FP advice during ANC and PNC visit have led to an increase in current use of contraceptives. However, these services have not motivated a decline in unmet need at the same pace. ATEs of these exposure variables are higher on current use of spacing method than that on limiting methods. There is not much difference in ATE of MH services utilization (including advice) on unmet need for FP by type of method.

When we compare average treatment effect on treated (ATT) and average treatment effect on untreated (ATU) (Table 3), considering all MH service utilization as exposure variable, we do not find differences between ATT and ATU. However, ATU is higher than ATT when exposure variable is ‘advised received on FP during ANC visit’. It suggests that the women who could not receive advice on FP during ANC, if they would have been advised the contraception use outcomes would be at higher level.

## Discussion

This study shows the continuity of different MH services and its effect on contraceptive use by comparing the utilization of succeeding services between two groups. The data from Uttar Pradesh has shown lower levels of service utilization with higher discontinuation of MH and FP services despite the high priority given to the RCH program[59]. These results show that prior service utilization promotes subsequent service utilization. The highest dropout is observed at the first level of care, i.e. ANC visit and later at institutional delivery level in rural Uttar Pradesh. If women go for institutional delivery there is high possibility that they will go for PNC. Use of modern contraceptives is 24% for those who availed all prior services (ANC, ID, PNC) in comparison to only 14% among those who had not received any service.

NRHM has increased the level of MH service utilization and contraceptive use through the RCH program in India[9]. The integrative approach is cost effective. The net savings and benefits accruing to the health system outweigh the initial costs [60]; It improves household incomes, enables greater investment in health, education and well-being[61].

Our findings reveals in rural Uttar Pradesh, women with low socio-economic profile were less likely to receive MH services, advice on FP during ANC and PNC visit. This is also supported by other research suggests that there is significant inequality among social groups in receiving advice on FP during ANC/PNC visits. The poor women are less likely to receive FP advice [34,58]. Such anomalies depend not only on the system’s efficiency but are also associated with cultural or social barriers. Therefore, study recommends that women with low socio-economic profile should be target to leverage service utilization with convergence of MH and FP.

The study finds that the utilization of critical MH services (any ANC, institutional delivery, PNC) encourages subsequent contraceptive use and reduces the unmet need for FP marginally. The increase in FP however could also be the result of a confounder such as integration of MH and FP services in India[38,62]. There is a need to strengthen this integration to reduce unmet need for contraceptive use. While comparing by type of methods, MH service utilization is more effective in increasing the use of spacing method and reducing unmet need for limiting method.

Research from developing countries indicates that FP advice recipients are more likely to use contraception than those who do not receive it[63]. A recent study from developed country among women found counseling regarding FP methods to be more effective in increasing contraceptive use[64]. Similar results are found in the present research that the advice on FP during ANC/PNC sessions has improved both contraceptive behavior outcomes. Advice during PNC is found to be more effective followed by, advice received during ANC to improve contraceptive behavior as found in previous studies [65–68]. This has a programmatic implications that advice on FP during PNC and ANC visits should be promoted with more efforts to increase contraceptive use and to reduce unmet need for FP. In the current Government program, Doctor, front line health workers including (accredited social health activist, auxiliary nurse midwives & anganwadi worker) are majorly responsible to provide FP advice or counseling during ANC and PNC period. With the present study we could not show the impact of quality and source of counseling. However, the advice by type of method has been explored.

While comparing between two outcome indicators contraceptive use in compare to unmet need for FP is found to be more influenced by counseling during MH services. As unmet need for FP depends on contraceptive use and demand for FP. Further, demand for FP is derived from the pregnancy intention in terms of limiting family size and birth spacing. If counseling on FP has generated more demand for FP (limiting and spacing) than the increase in use of contraceptives, unmet need may not reduce with same pace as contraception use has increased.

In a low-resource setting such as Uttar Pradesh, the health system approach to improving ANC/PNC services should be prioritized with more effective advice on FP to reduce unintended births. FP advice is part of the routine PNC services and the opportunity to receive information and support for FP use should be available to all women irrespective of their social background.

Although findings of this study offer important insights about MH outcomes and its association with contraceptive use and unmet need for FP, these results must be interpreted in the light of study limitations. In DLHS-III, the information on advice on FP during ANC/PNC session was obtained from women at the time of survey and thus responses of advice might be affected by recall bias. The analysis was also restricted only to the last birth that took place in the three years preceding the survey. The study could not examine the quality of PNC services offered in public/private health facilities. Nevertheless, our findings hold important implications for planning and implementation of programs.

## References

[1] Singh S, Darroch JE, Ashford LS, Vlassoff M (2009) Adding it up: the costs and benefi ts of investing in family planning and maternal and newborn health. New York: Guttmacher Institute and United Nations Population Fund.

[2] Liu L, Becker S, Tsui A, Ahmed S (2008) Three methods of estimating births averted nationally by contraception. Popul Stud (Camb) 62: 191–210. 10.1080/00324720801897796 18587694

[3] Ahmed S, Li Q, Liu L, Tsui AO (2012) Maternal deaths averted by contraceptive use: an analysis of 172 countries. The Lancet 380: 111–125. 10.1016/S0140-6736(12)60478-4 22784531

[4] Waddington C, Egger D (2008) Integrated Health Services—What and why? Geneva World Health Organization.

[5] Ringheim K, Gribble J, Foreman M (2011) Integrating Family Planning and Maternal and Child Health Care: Saving Lives, Money, and Time. Policy Brief. Washington: Population Reference Bureau.

[6] MoHFW (1997) Reproductive and Child Health Programme: Schemes for Implementation. New Delhi: Ministry of Health & Family Welfare, Government of India.

[7] MoHFW (2000) National Population Policy 2000. New delhi: MInistry of Health and Family Welfare, Government of India.

[8] MoHFW (2002) National Health Policy 2002. New Delhi: Ministry of Health & Family Welfare, Government of India.

[9] MoHFW (2005) National Rural Health Mission (2005–2012): Mission Document. New Delhi: Ministry of Health & Family Welfare, Government of India.

[10] IIPS, MI (2007) National Family Health Survey (NFHS-3), 2005–2006. Mumbai: IIPS.

[11] Nikiéma B, Beninguisse G, Haggerty JL (2009) Providing information on pregnancy complications during antenatal visits: unmet educational needs in sub-Saharan Africa. Health Policy and Planning 24: 367–376. 10.1093/heapol/czp017 19401360

[12] Renkert S, Nutbeam D (2001) Opportunities to improve maternal health literacy through antenatal education: an exploratory study. Health Promotion International 16: 381–388. 11733456 10.1093/heapro/16.4.381

[13] WHO (2003) Working with individuals, families and communities to improve maternal and new born health Geneva 27, Switzerland: World Health Organization

[14] Pembe AB, Urassa DP, Carlstedt A, Lindmark G, Nystrom L, et al (2009) Rural Tanzanian women’s awareness of danger signs of obstetric complications. BMC Pregnancy Childbirth 9: 12 10.1186/1471-2393-9-12 19323836 PMC2667432

[15] Magoma M, Requejo J, Campbell OM, Cousens S, Filippi V (2010) High ANC coverage and low skilled attendance in a rural Tanzanian district: a case for implementing a birth plan intervention. BMC Pregnancy Childbirth 10: 13 10.1186/1471-2393-10-13 20302625 PMC2850322

[16] Magoma M, Requejo J, Merialdi M, Campbell OM, Cousens S, et al (2011) How much time is available for antenatal care consultations? Assessment of the quality of care in rural Tanzania. BMC Pregnancy Childbirth 11: 64 10.1186/1471-2393-11-64 21943347 PMC3195209

[17] Levitt C, Shaw E, Wong S, Kaczorowski J, Springate R, et al (2004) Systematic review of the literature on postpartum care: selected contraception methods, postpartum Papanicolaou test, and rubella immunization. Birth 31: 203–212. 15330883 10.1111/j.0730-7659.2004.00306.x

[18] Zerai A, Tsui AO (2001) The relationship between prenatal care and subsequent modern contraceptive use in Bolivia, Egypt and Thailand. Afr J Reprod Health 5: 68–82. 12471915

[19] Hotchkiss DR, Magnani RJ, Rous JJ, Azelmat M, Mroz TA, et al (1999) The effects of maternal-child health service utilization on subsequent contraceptive use in Morocco. J Biosoc Sci 31: 145–165. 10333649 10.1017/s0021932099001455

[20] Hotchkiss D, Rous J, Seiber E, Berruti A (2005) Is Maternal and Child Health Service use a Causal Gateway to Subsequent Contraceptive use?: A Multi-Country Study. Population Research and Policy Review 24: 543–571.

[21] Seiber EE, Hotchkiss DR, Rous JJ, Berruti AA (2005) Maternal and child health and family planning service utilization in Guatemala: implications for service integration. Soc Sci Med 61: 279–291. 15893045 10.1016/j.socscimed.2004.11.068

[22] Ahmed S, Mosley WH (2002) Simultaneity in the use of maternal-child health care and contraceptives: evidence from developing countries. Demography 39: 75–93. 11852841 10.1353/dem.2002.0001

[23] WHO (2004) Reproductive Health Strategy to Accelerate Progress Towards the Attainment of International Development Goals and Targets. Geneva: World Health Organization. 10.1016/s0968-8080(05)25166-2 16035592

[24] Matthews Z, Mahendra S, Kilaru A, Ganapathy S (2001) Antenatal care, careseeking and morbidity in rural Karnataka, India: results of a prospective study. Asia-Pacific Population Journal 16: 11–28.

[25] Pallikadavath S, Foss M, Stones RW (2004) Antenatal care: provision and inequality in rural north India. Soc Sci Med 59: 1147–1158. 15210087 10.1016/j.socscimed.2003.11.045

[26] Ram F, Singh A (2006) Is antenatal care effective in improving maternal health in rural uttar pradesh? Evidence from a district level household survey. J Biosoc Sci 38: 433–448. 16762083 10.1017/S0021932005026453

[27] Sugathan KS, Mishra V, Retherford RD (2001) Promoting Institutional Delievries in Rural India: The Role of Antenatal-Care Services. Mumbai: International Institute for Population Sciences, Mumbai, India and East-West Center, Population and Health Studies, Honolulu, Hwaii, USA 20 20.

[28] Birungi H, Onyango-Ouma W (2006) Acceptability and Sustainability of the WHO Focused Antenatal Care package in Kenya. Washington, DC: Population Council, Frontiers in Reporductive Health.

[29] Sines E, Syed U, Wall S, Worley H (2007) Postnatal Care: A Critical Opportunity to Save Mothers and Newborns. Washington, DC: Population Reference Bureau.

[30] Dhakal S, Chapman GN, Simkhada PP, van Teijlingen ER, Stephens J, et al (2007) Utilisation of postnatal care among rural women in Nepal. BMC Pregnancy Childbirth 7: 19 17767710 10.1186/1471-2393-7-19 PMC2075509

[31] Anwar ATMI, Killewo J, Chowdhury M-E-EK, Sushil, Dasgupta K (2004) Bangladesh: Inequalities in Utilization of Maternal Health Care Services-Evidence from MATLAB. Washington, DC: The World Bank.

[32] Jat TR, Ng N, San Sebastian M (2011) Factors affecting the use of maternal health services in Madhya Pradesh state of India: a multilevel analysis. Int J Equity Health 10: 59 10.1186/1475-9276-10-59 22142036 PMC3283453

[33] Mistry R, Galal O, Lu M (2009) "Women’s autonomy and pregnancy care in rural India: a contextual analysis". Soc Sci Med 69: 926–933. 10.1016/j.socscimed.2009.07.008 19656604

[34] Singh A, Padmadas SS, Mishra US, Pallikadavath S, Johnson FA, et al (2012) Socio-economic inequalities in the use of postnatal care in India. PLoS One 7: e37037 10.1371/journal.pone.0037037 22623976 PMC3356397

[35] Lopez LM, Hiller JE, Grimes DA, Chen M (2012) Education for contraceptive use by women after childbirth. Cochrane Database Syst Rev 8: CD001863 10.1002/14651858.CD001863.pub3 22895923

[36] Briggs CJ, Capdegelle P, Garner P (2001) Strategies for integrating primary health services in middle- and low-income countries: effects on performance, costs and patient outcomes. Cochrane Database Syst Rev: CD003318 11687187 10.1002/14651858.CD003318

[37] Huntington D, Aplogan A (1994) The integration of family planning and childhood immunization services in Togo. Stud Fam Plann 25: 176–183. 7940622

[38] Taylor CE, Parker RL (1987) Integrating PHC services: evidence from Narangwal, India. Health Policy and Planning 2: 150–161.

[39] IIPS (2010) District Level Household and Facility Survey (DLHS-3), 2007–08. In: IIPS, editor. Mumbai: International Institute for Population Sciences 10.1371/journal.pone.0071584

[40] India Go (2011) Census of India 2011: Provisional Population Totals, Paper 1 of 2011, India, Series 1. New Delhi: Office of Registrar General & Census Commissioner, Government of India (GOI).

[41] RGI (2011) Sample Registration System (SRS), Statistical Report 2011. New Delhi: Office of the Registrar General, Governemnt of India.

[42] Commission P (2011) India Human Development Reprot 2011, Towards Social Inclusion. New Delhi: Planning Commission, Government of India 568 p.

[43] Howe LD, Hargreaves JR, Gabrysch S, Huttly SR (2009) Is the wealth index a proxy for consumption expenditure? A systematic review. J Epidemiol Community Health 63: 871–877. 10.1136/jech.2009.088021 19406742

[44] Vyas S, Kumaranayake L (2006) Constructing socio-economic status indices: how to use principal components analysis. Health Policy Plan 21: 459–468. 17030551 10.1093/heapol/czl029

[45] Rosenbaum PR, Rubin DB (1985) Constructing a Control Group Using Multivariate Matched Sampling Methods That Incorporate the Propensity Score. The American Statistician 39: 33–38.

[46] Stuart EA (2010) Matching methods for causal inference: A review and a look forward. Stat Sci 25: 1–21. 20871802 10.1214/09-STS313 PMC2943670

[47] Rosenbaum PR, Rubin DB (1983) The central role of the propensity score in observational studies for causal effects. Biometrika 70: 41–55.

[48] Rubin DB, Thomas N (1996) Matching using estimated propensity scores: relating theory to practice. Biometrics 52: 249–264. 8934595

[49] Williamson E, Morley R, Lucas A, Carpenter J (2012) Propensity scores: from naive enthusiasm to intuitive understanding. Stat Methods Med Res 21: 273–293. 10.1177/0962280210394483 21262780

[50] Guha M, Baschieri A, Bharat S, Bhatnagar T, Sane SS, et al (2012) Risk reduction and perceived collective efficacy and community support among female sex workers in Tamil Nadu and Maharashtra, India: the importance of context. Journal of Epidemiology and Community Health 66: ii55–ii61. 10.1136/jech-2011-200562 22760217

[51] StataCorp (2009) Stata Statistical Software: Release 12. College Station, TX: StataCorp LP.

[52] Bhattacharjee P, Prakash R, Pillai P, Isac S, Haranahalli M, et al (2013) Understanding the role of peer group membership in reducing HIV-related risk and vulnerability among female sex workers in Karnataka, India. AIDS Care 25: S46–S54. 10.1080/09540121.2012.736607 23745630 PMC4003574

[53] Yadav D, Ramanathan S, Goswami P, Ramakrishnan L, Saggurti N, et al (2013) Role of community group exposure in reducing sexually transmitted infection-related risk among female sex workers in India. PLoS One 8: e78361 10.1371/journal.pone.0078361 24205210 PMC3813446

[54] Juneja S, Rao Tirumalasetti V, Mishra RM, Sethu S, Singh IR (2013) Impact of an HIV Prevention Intervention on Condom Use among Long Distance Truckers in India. AIDS and Behavior 17: 1040–1051. 10.1007/s10461-012-0314-y 23008122 PMC3586141

[55] Dixit P, Dwivedi LK, Ram F (2013) Strategies to Improve Child Immunization via Antenatal Care Visits in India: A Propensity Score Matching Analysis. PLoS ONE 8: e66175 23824555 10.1371/journal.pone.0066175 PMC3688852

[56] Lechner M (2002) Some practical issues in the evaluation of heterogeneous labour market programmes by matching methods. Journal of the Royal Statistical Society: Series A (Statistics in Society) 165: 59–82.

[57] Oakes JM, Kaufman JS, editors (2006) Methods in Social Epidemiology. San Francisco, CA: John Wiley & Sons.

[58] Singh A, Pallikadavath S, Ram F, Ogollah R (2012) Inequalities in advice provided by public health workers to women during antenatal sessions in rural India. PLoS One 7: e44931 10.1371/journal.pone.0044931 23028688 PMC3444494

[59] Yadav D (2012) A Study of synergies between maternal-child health and family planning services utilization in rural Uttar Pradesh. Mumbai: International Institute for Population Sciences.

[60] Rodriguez-Barocio R, Garcia-Nunez J, Urbina-Funtes M, Wulf D (1980) Fertility and Family Planning in Mexico. International Family Planning Perspectives 6: 2–9.

[61] Joshi S, Schultz TP (2007) Family Planning as an Investment in Development: Evaluation of a Program’s Consequences in Matlab, Bangladesh. New Haven, CT: Economic Growth Center, Yale University.

[62] Toth C (2008) The Right Message-to the Right People-at the Right Time.

[63] Do M, Hotchkiss D (2013) Relationships between antenatal and postnatal care and post-partum modern contraceptive use: evidence from population surveys in Kenya and Zambia. BMC Health Serv Res 13: 6 10.1186/1472-6963-13-6 23289547 PMC3545900

[64] Lee JK, Parisi SM, Akers AY, Borrero S, Schwarz EB (2011) The impact of contraceptive counseling in primary care on contraceptive use. Journal of General Internal Medicine 26: 731–736. 10.1007/s11606-011-1647-3 21301983 PMC3138576

[65] Koenig MA, Rob U, Khan MA, Chakraborty J, Fauveau V (1992) Contraceptive use in Matlab, Bangladesh in 1990: levels, trends, and explanations. Stud Fam Plann 23: 352–364. 1293859

[66] Glasier AF, Logan J, McGlew TJ (1996) Who gives advice about postpartum contraception? Contraception 53: 217–220. 8706439 10.1016/0010-7824(96)00040-6

[67] Ozvaris SB, Akin A, Yildiran M (1997) Acceptability of postpartum contraception in Turkey. Advances in Contraceptive Delivery Systems 13: 63–71.

[68] Fagan EB, Rodman E, Sorensen EA, Landis S, Colvin GF (2009) A survey of mothers’ comfort discussing contraception with infant providers at well-child visits. South Med J 102: 260–264. 10.1097/SMJ.0b013e318197fae4 19204612

